# Interaction Effects of Nitrogen Rates and Forms Combined With and Without Zinc Supply on Plant Growth and Nutrient Uptake in Maize Seedlings

**DOI:** 10.3389/fpls.2021.722752

**Published:** 2021-12-09

**Authors:** Yanfang Xue, Wei Yan, Yingbo Gao, Hui Zhang, Liping Jiang, Xin Qian, Zhenling Cui, Chunyan Zhang, Shutang Liu, Huimin Wang, Zongxin Li, Kaichang Liu

**Affiliations:** ^1^National Engineering Laboratory of Wheat and Maize, Key Laboratory of Biology and Genetic Improvement of Maize in Northern Yellow-Huai Rivers Plain, Ministry of Agriculture, Maize Research Institute, Shandong Academy of Agricultural Sciences, Jinan, China; ^2^College of Resources and Environment, Qingdao Agricultural University, Qingdao, China; ^3^College of Resources and Environment, China Agricultural University, Beijing, China; ^4^Linyi Academy of Agricultural Sciences, Linyi, China

**Keywords:** maize, nitrogen rates and forms, zinc supply, interaction effects, nutrient uptake, root morphology

## Abstract

Previous studies have shown that zinc (Zn) accumulation in shoot and grain increased as applied nitrogen (N) rate increased only when Zn supply was not limiting, suggesting a synergistic effect of N on plant Zn accumulation. However, little information is available about the effects of different mineral N sources combined with the presence or absence of Zn on the growth of both shoot and root and nutrient uptake. Maize plants were grown under sand-cultured conditions at three N forms as follows: NO_3_^–^ nutrition alone, mixture of NO_3_^–^/NH_4_^+^ with molar ratio of 1:1 (recorded as mixed-N), and NH_4_^+^ nutrition alone including zero N supply as the control. These treatments were applied together without or with Zn supply. Results showed that N forms, Zn supply, and their interactions exerted a significant effect on the growth of maize seedlings. Under Zn-sufficient conditions, the dry weight (DW) of shoot, root, and whole plant tended to increase in the order of NH_4_^+^ < NO_3_^–^ < mixed-N nutrition. Compared with NH_4_^+^ nutrition alone, mixed-N supply resulted in a 27.4 and 28.1% increase in leaf photosynthetic rate and stomatal conductance, which further resulted in 35.7 and 33.5% of increase in shoot carbon (C) accumulation and shoot DW, respectively. Furthermore, mixed-N supply resulted in a 19.7% of higher shoot C/N ratio vs. NH_4_^+^ nutrition alone, which means a higher shoot biomass accumulation, because of a significant positive correlation between shoot C/N ratio and shoot DW (*R*^2^ = 0.682^***^). Additionally, mixed-N supply promoted the greatest root DW, total root length, and total root surface area and synchronously improved the root absorption capacity of N, iron, copper, manganese, magnesium, and calcium. However, the above nutrient uptake and the growth of maize seedlings supplied with NH_4_^+^ were superior to either NO_3_^–^ or mixed-N nutrition under Zn-deficient conditions. These results suggested that combined applications of mixed-N nutrition and Zn fertilizer can maximize plant growth. This information may be useful for enabling integrated N management of Zn-deficient and Zn-sufficient soils and increasing plant and grain production in the future.

## Introduction

Nitrogen (N) is the most abundant mineral nutrient element in plants and plays an essential role in plant growth and development. Nitrate (NO_3_^–^-N) and ammonium (NH_4_^+^-N) are predominant forms of inorganic N in soil, both of which can be absorbed by plants directly (Marschner, 2011). The preferred N form varies among plant species and among genotypes of individual plant species, environmental conditions, and developmental stages (Cramer and Lewis, 1993; Zhao et al., 2013). Maize usually grows in well-aerated dryland soils, where NO_3_^–^ is the major source of N due to soil nitrification (Fang et al., 2006). Some studies showed the enhanced growth of maize under NO_3_^–^ nutrition compared with NH_4_^+^ nutrition (Cramer and Lewis, 1993; Schortemeyer et al., 1997). Others showed that NH_4_^+^ uptake may be an important strategy for maize plants to take up sufficient quantities of N for growth compared with NO_3_^–^ nutrition (Gu et al., 2013; George et al., 2016; Wang et al., 2019; Zhang H. Q. et al., 2019). For most plants, a mixture of both NO_3_^–^ and NH_4_^+^ is superior to either NO_3_^–^ or NH_4_^+^ sources alone, possibly due to improved cytokinin transportation from root to shoot, stimulated leaf growth, maintained rhizosphere pH, and increased nutrient uptake rates, such as N, phosphorus (P), copper (Cu), and iron (Fe) (Gentry and Below, 1993; Marschner, 2011; Cao et al., 2018; Wang et al., 2019).

Zinc (Zn) is an essential microelement for plant growth. Zinc plays a vital role in many essential cellular functions and metabolic pathways and is important to the normal health and reproduction of plants, animals, and humans (Welch and Graham, 2004). It is estimated that more than 30% of the agricultural soils in the world are prone to Zn deficiency (Alloway, 2008). According to a national soil survey, China has nearly 0.63 billion hm^2^ of soil, accounting for 45.7% of the farmland, is subject to Zn deficiency, primarily calcareous soil in Northern China (Liu, 1996). Although Zn is an essential micronutrient for plant growth, Zn input has received much less attention than N, P, or irrigation during the Green Revolution (Tilman et al., 2002; Mueller et al., 2012). Therefore, the application of Zn fertilizers is necessary in such soils to ensure cereal yield and grain Zn concentration (Cakmak, 2008).

Maize is one of the world’s major crops and is expected to contribute increasingly to human and animal nutrition and to energy production. Maize is sensitive to Zn deficiency (Wang and Jin, 2007). Zn-deficient symptoms, such as stunted and chlorotic plants, are often observed in maize plants growing on calcareous soil in the field (Alloway, 2008). Many studies have demonstrated that grain yield of maize was increased by the application of Zn fertilizer to Zn-deficient soils. The positive effects of Zn supply are related to the increased photosynthesis rates, chlorophyll content in leaves, and increased kernel number and weight in the apical benefited from increased pollen viability at the tasseling stage under field conditions (Potarzycki, 2010; Wang et al., 2012; Liu et al., 2016, 2020).

Previous results from greenhouse conditions showed that increasing N supply did not improve Zn accumulation in grain and shoot of wheat in case of low soil Zn availability. Otherwise, increasing N supply substantially improved Zn concentration and accumulation of both grain and shoot in wheat when Zn supply was not limited, showing a synergistic effect of N fertilization on Zn accumulation (Kutman et al., 2010, 2011a,b). Furthermore, our results obtained from field conditions also showed positive effects of N application on the root uptake, root-to-shoot translocation, and remobilization of Zn in wheat and maize grown in Zn-sufficient soils (Xue et al., 2012, 2014, 2019). However, little information is available about the effects of different mineral N sources combined with the presence or absence of Zn on the growth of both shoot and root and nutrient uptake.

This study aimed to determine how the different N forms affected the growth of shoot and root, root morphological traits, and nutrient uptake of maize seedlings grown with and without Zn supply under greenhouse conditions. This information may be useful for enabling integrated N management of Zn-deficient and Zn-sufficient soils and increasing plant and grain production in the future.

## Materials and Methods

### Experimental Procedures

Seeds of hybrid “Denghai 605” were surface-sterilized by soaking in 10% (v/v) H_2_O_2_ for 30 min, rinsed thoroughly in deionized water, soaked in saturated CaSO_4_ for 5 h, then transferred to germinate on filter paper in the dark at 25°C for 2 days. Initially, five uniformly germinated seeds were sown in a plastic pot filled with 5 kg of quartz sand (diameter between 0.25 and 0.50 mm) and later thinned to three plants per pot. Quartz sand was pretreated by soaking in acid (5 N HCl) for 18 h and was thoroughly washed with distilled water until pH was neutral. Each treatment had seven replicates arranged in a completely randomized design. Their places were changed frequently inside a glasshouse with day/night temperature of 25/18°C. Plants were supplied with nutrient solution after germination according to Yuan et al. (2011). The nutrient solution contained 0.75 mmol L^–1^ K_2_SO_4_, 0.65 mmol L^–1^ MgSO_4_⋅7H_2_O, 0.25 mmol L^–1^ KH_2_PO_4_, 10 μmol L^–1^ H_3_BO_3_, 1 μmol L^–1^ MnSO_4_⋅H_2_O, 0.5 μmol L^–1^ CuSO_4_⋅5H_2_O, 0.05 μmol L^–1^ Na_2_Mo_7_O_4_⋅2H_2_O, and 0.1 mmol L^–1^ Fe-EDTA. Nitrogen was supplied in 4 mmol L^–1^ with three different N forms as follows: NO_3_^–^ nutrition alone, mixture of NO_3_^–^/NH_4_^+^ with molar ratio of 1:1 (recorded as mixed-N), and NH_4_^+^ nutrition alone including zero N supply as the control (recorded as N0). These treatments were applied together without (Zn0), or with 2 μmol L^–1^ ZnSO_4_⋅7H_2_O (Zn1). NH_4_^+^-N and NO_3_^–^-N were applied in the form of (NH_4_)_2_SO_4_ and Ca(NO_3_)_2_⋅4H_2_O, respectively. Furthermore, CaCl_2_⋅2H_2_O was added to balance calcium in the solution that contained ammonium and the control treatments as well. The solution pH was adjusted to 6.0. The pots were watered every 2 days using 400 ml of treated nutrient solution.

### Photosynthetic Parameter Measurements

Photosynthetic parameters including net photosynthetic rate (*P*_*n*_), transpiration rate (*T*_*r*_), stomatal conductance (*g*_*s*_), and intercellular CO_2_ concentration (*C*_*i*_) were measured using a portable Li-6400 photosynthesis system (LI-COR Inc., Lincoln, NE, United States). The youngest fully expanded leaves at 30 days after N and Zn treatments were selected for measurement between 09:00 and 11:00. The photosynthetically active radiation, temperature, and CO_2_ concentration during measurement collection were set at 600 μmol m^–2^ s^–1^, 25°C, and 380 μmol mol^–1^, respectively. The index of chlorophyll content (SPAD value) in the youngest fully expanded leaves was determined using portable chlorophyll meter SPAD-502 Plus (SPAD, Minolta Camera Co., Ltd., Osaka, Japan). There were four biological replicates for each treatment.

### Mineral Elements

After photosynthetic parameter measurements, plants were harvested for 30 days after N and Zn treatments. The fresh plant samples were divided into shoots and roots. The roots were washed in 0.5 mM CaSO_4_ solution for 10 min, and shoots were then rinsed with ultra-pure water and dried at 65°C until constant weight. The dry weight (DW) of shoot and root was used to calculate Zn efficiency (ZE) (the ratio of DW of shoot or root without Zn supply to that with Zn supply ×100), which has been used for screening various genotypes of cereals and other crops (Karim et al., 2012). The dried samples were ground with a stainless steel grinder and then digested using an acid mixture of HNO_3_–H_2_O_2_ in a microwave accelerated reaction system (CEM Corp., Matthews, NC, United States). The concentrations of Zn, Fe, manganese (Mn), Cu, calcium (Ca), magnesium (Mg), P, and K (potassium) in the digested solutions were determined by inductively coupled plasma atomic emission spectroscopy (ICP-AES, Optima 7300 DV, PerkinElmer, Waltham, MA, United States). Two blanks and a certified reference material (IPE126, Wageningen University, Netherlands) were included in each batch of digestion to ensure analytical quality. The concentrations of carbon (C) and N in samples were determined using a CN analyzer (vario Macro cube, Elementar, Hanau, Germany). There were four biological replicates for each treatment.

### Leaf Soluble Protein Content

About 30 days after N and Zn treatments, the youngest fully expanded leaves from another batch of samples with three biological replicates for each treatment were frozen in liquid N_2_ and crushed with a tissue homogenizer. The crushed samples were dissolved in pre-cooled phosphate buffer with pH 7.8 and made up to a final volume of 1,000 ml. The concentration of protein extracts was determined by a colorimetric method as described by Bradford (1976) using protein assay dye Coomassie Brilliant Blue G-250 (Amresco, Athens, GA, United States). The absorbance was determined at 595 nm.

### Root Morphological Parameters

After the youngest fully expanded leaves were removed for determination of soluble protein, root samples were washed with tap water and kept at −20°C before an image was captured using an optical scanner (Epson, Nagano, Japan). The root traits were determined by analysis of images using the WinRHIZO software (Regent Instrument, Quebec, QC, Canada). There were three biological replicates for each treatment. After completing the root scan, these roots were not used for nutrient analysis due to the possibility of nutritional loss during the scanning process.

### Statistical Analysis

ANOVA was performed using the Data Processing System (DPS) version 9.50 statistical software (Tang and Zhang, 2013). Means were compared using Fisher’s protected least significant difference (LSD) tests. Differences were considered significant at *p* < 0.05. Linear regression analysis was employed to identify the correlation between shoot C/N ratio and shoot DW using the SAS software (SAS 8.0, SAS Institute Inc., Cary, NC, United States).

## Results

### Dry Weight of Shoot and Root and the Ratio of Root-to-Shoot

Our data revealed significant effects of the Zn and N treatments on the DW of the shoot, root, and whole plants and the root-to shoot ratios (R/S) of 30-day-old maize plants grown under greenhouse conditions (Table 1). The interactions between Zn and N treatments were also significant for the DW of the shoot, root, and whole plants. Under a Zn-deficient condition, plants grown with NH_4_^+^ nutrition produced significantly greater shoot DW than with mixed N nutrition, whereas NO_3_^–^ produced an intermediate shoot DW (Table 1). Plants grown with NH_4_^+^ also produced slightly higher root DW (approximately 27%) but not significantly different from NO_3_^–^ and mixed N supply. However, under a Zn-sufficient condition, plants grown with NO_3_^–^ supply and mixed-N supply produced significantly greater shoot DW than with NH_4_^+^ supply (Table 1). Plants grown with mixed-N nutrition produced significantly higher root DW than with either N form alone. Compared with nil N supply, N supply resulted in a 2.6- to 3.7-fold increase in shoot DW and 0.2- to 0.6-fold increase in root DW under Zn-deficient conditions. Under Zn-sufficient conditions, N supply resulted in a 3.9- to 4.8-fold increase in shoot DW and 0.5- to 1.2-fold increase in root DW. Consequently, although the R/S was not significantly affected by the three N forms, N supply vs. nil N supply remarkably reduced the R/S from 81.9 to 27.2% and from 71.1 to 22.4% in response to Zn-deficient and Zn-sufficient conditions.

**TABLE 1:** Dry weight (DW) of shoot, root, and whole plant, Zn efficiency (ZE), and root-to-shoot ratio (R/S) of 30-day-old maize seedlings cultured with nil N (N0), NO_3_^–^, mixed-N, and NH_4_^+^ nutrition under sand culture conditions without (Zn0) and with Zn (Zn1) supply.

N treatment	Shoot DW (g pot^–1^)		ZE (%)	Root DW (g pot^–1^)		ZE (%)	Whole plant (g pot^–1^)		ZE (%)	R/S (%)	
	Zn0	Zn1		Zn0	Zn1		Zn0	Zn1		Zn0	Zn1
N0	0.91e	1.12e	82.0a	0.75d	0.80d	96.9a	1.66e	1.91e	87.6a	81.9a	71.1b
NO_3_^–^	3.46dc	6.44a	53.9b	0.91dc	1.35b	69.5b	4.38dc	7.78b	56.4b	26.4c	20.8c
Mixed-N	3.24d	7.32a	44.6b	0.92dc	1.77a	54.0b	4.16d	9.09a	46.2b	28.4c	24.2c
NH_4_^+^	4.33c	5.48b	81.4a	1.17bc	1.18bc	98.1a	5.50c	6.66b	83.8a	26.8c	22.1c
Source of variation											
Zn treatment (Zn)		***			***			***			*
N treatment (N)		***			***			***			***
Zn × N		***			**			***			NS

Values followed by different lowercase letters in columns represent significant differences among the N and Zn treatments. The symbols *, **, and *** indicate significant difference at 0.05, 0.01, and 0.001 level, respectively. NS, no significant difference.

Compared with no Zn supply, Zn supply resulted in 22.4–125.9, 0.5–91.8, and 15.3–118.4% greater DW of the shoot, root, and whole plant, respectively, with the increase being the highest with mixed-N nutrition and following by NO_3_^–^ supply. However, ZE was significantly higher with NH_4_^+^ nutrition compared with NO_3_^–^ and mixed-N supply, showing a greater ability to resist Zn deficiency (Table 1).

### SPAD Readings and Soluble Protein Contents of Newly Developed Leaf

Two-way ANOVA revealed significant effects of the N treatments on the leaf SPAD values and soluble protein content, and significant effects of the Zn supply only on leaf SPAD values. The interaction between N treatments and Zn supply was also significant for leaf soluble protein content (Supplementary Table 1).

**FIGURE 1:**
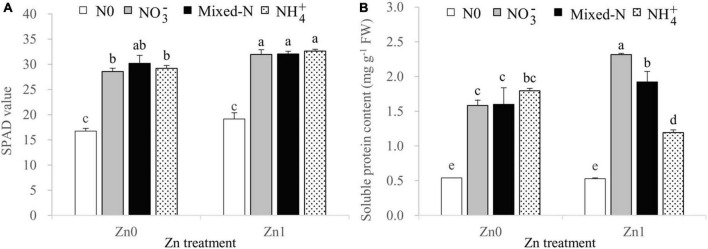
SPAD readings **(A)** and soluble protein contents **(B)** of newly developed leaf of 30-day-old maize seedlings cultured with nil N (N0), NO_3_^–^, mixed-N, and NH_4_^+^ nutrition under sand culture conditions without (Zn0) and with Zn (Zn1) supply. Error bars represent the standard error of the mean (*n* = 4). Significant differences at *p* < 0.05 are shown with different letters.

Compared with no N supply, the three N forms significantly increased the leaf SPAD readings and leaf soluble protein contents (Figures 1A,B). There were no significant differences in the leaf SPAD readings among the three N forms, regardless of Zn supply (Figure 1A). The leaf soluble protein contents increased gradually in the order of NH_4_^+^ < mixed-N < NO_3_^–^ nutrition in the presence of Zn supply. However, these values were not significantly affected by the different N forms under Zn-deficient conditions (Figure 1B).

### Leaf Photosynthetic Parameters

**FIGURE 2:**
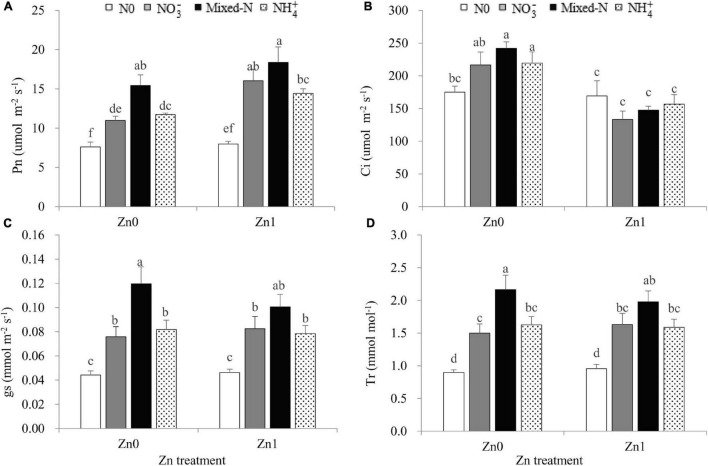
Leaf photosynthetic parameters including *P_*n*_* **(A)**, *C_*i*_* **(B)**, *g_*s*_* **(C)** and *T_*r*_* **(D)** of newly developed leaf of 30-d-old maize seedlings cultured with nil N (N0), NO_3_^–^, mixed-N and NH_4_^+^ nutrition under sand culture conditions without (Zn0) and with Zn (Zn1) supply. Error bars represent the standard error of the mean (*n* = 4). Significant differences at *P* < 0.05 are shown with different letters. *P_*n*_*, photosynthetic rate; *C_*i*_*, intercellular CO_2_ concentration; *g_*s*_*, conductance to H_2_O; *T_*r*_*, transpiration rate.

Two-way ANOVA revealed significant effects of the N treatments on the leaf *P*_*n*_, *g*_*s*_, and *T*_*r*_ and significant effects of the Zn supply on *P*_*n*_ and C*_*i*_* (Supplementary Table 1). The interaction between N treatments and Zn supply was significant only for leaf *C*_*i*_ (Supplementary Table 1). Compared with nil N supply, three N forms significantly increased the leaf *P*_*n*_, *g*_*s*_, and *T*_*r*_, irrespective of Zn supply (Figures 2A,C,D). Plants grown with mixed-N nutrition had significantly higher leaf *P*_*n*_, *g*_*s*_, and *T*_*r*_ than those supplied with either N form alone under Zn-deficient conditions. Similar results were also observed under Zn-sufficient conditions, although less pronounced (Figures 2A,C,D). Furthermore, Zn supply resulted in a 5.1–46.0% increase in leaf *P*_*n*_ (Figure 2A) and a 3.3–39.0% decrease in leaf *C*_*i*_ in comparison with no Zn supply (Figure 2B).

### Root Length as Affected by N and Zn Supply

**FIGURE 3:**
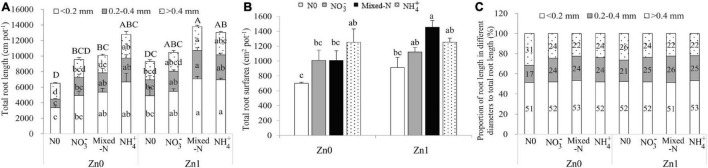
Root length **(A)**, root surface area **(B)** and the proportions of root length in different diameters to total root length **(C)** of 30-d-old maize seedlings cultured with nil N (N0), NO_3_^–^, mixed-N and NH_4_^+^ nutrition under sand culture conditions without (Zn0) and with Zn (Zn1) supply. Error bars represent the standard error of the mean (*n* = 3). Significant differences at *P* < 0.05 are shown with different letters.

Roots were divided into three categories based on root diameter: fine roots (diameter < 0.2 mm), medium-sized roots (diameter between 0.2 and 0.4 mm), and thick roots (diameter >0.4 mm). Two-way ANOVA revealed significant effects of the N treatments on the fine root length (FRL), medium-sized root length (MRL), thick root length, and total root length (TRL). Significant effects of Zn supply on those parameters were observed with an exception of thick roots. However, the interaction between N treatments and Zn supply was not significantly different for these parameters (Supplementary Table 2). Compared with nil N supply, N supply generally improved the FRL, MRL, and thick root length and finally increased the TRL although there was no significant difference between nil N and NO_3_^–^ nutrition irrespective of Zn supply. Among the three N forms, NH_4_^+^ supply produced the highest FRL, MRL, and thick roots and finally resulted in the highest TRL in the absence of Zn supply. Mixed-N supply resulted in the greatest TRL including the root length in different diameters in the presence of Zn supply. Compared with no Zn supply, Zn supply resulted in a 2.1–42.8% increase in TRL, with a significant increase with mixed-N supply (Figure 3A). Similar results were also observed for the total root surface area (Figure 3B).

The proportion of FRL to TRL was not affected by N and Zn supply and averaged 52.2%. The proportion of MRL to TRL (ranging from 17.1 to 26.2%) tended to increase, whereas the thick root length to TRL (ranging from 22.0 to 31.5%) tended to decrease with N supply compared with no N supply (Figure 3C).

### Nitrogen, C, and Zn Concentration and Accumulation of Shoot and Root

**FIGURE 4:**
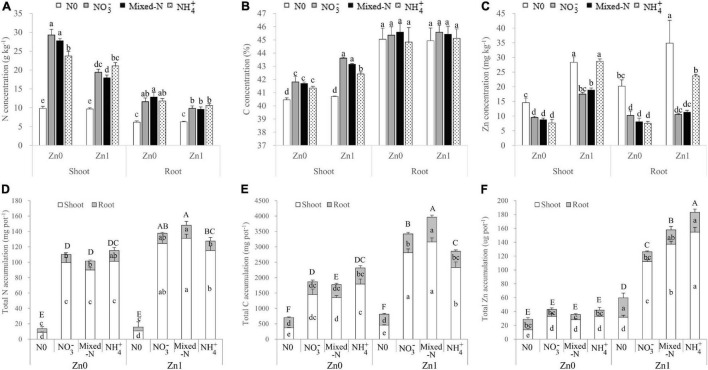
Nitrogen, C, and Zn concentration **(A–C)** and accumulation **(D–F)** of shoot and root of 30-day-old maize seedlings cultured with nil N (N0), NO_3_^–^, mixed-N, and NH_4_^+^ nutrition under sand culture conditions without (Zn0) and with Zn (Zn1) supply. Error bars represent the standard error of the mean (*n* = 4). Significant differences at *p* < 0.05 are shown with different letters.

According to the results of two-way ANOVA, N and Zn treatments had significant effects on the N concentration and accumulation of shoots and roots, with the exception of a non-significant effect of Zn treatments on root N accumulation. The interactions between N and Zn treatments were also significant for the shoot N concentration and accumulation (Supplementary Table 3). Compared with nil N supply, three N forms substantially increased the N concentration in both shoot and root. The root N concentration was not significantly different among the three N forms irrespective of Zn supply (Figure 4A). Among the three N forms, shoot N concentration gradually decreased in the order NH_4_^+^ > NO_3_^–^ > mixed-N nutrition in the presence of Zn supply. However, shoot N concentration was significantly lower in plants with NH_4_^+^ nutrition than NO_3_^–^ and mixed-N nutrition in the absence of Zn supply (Figure 4A). Generally, the change in shoot N concentration was opposite to that of shoot DW accumulation, regardless of Zn supply in response to different N forms. Because the increase in shoot DW was greater than the decrease in shoot N concentration, the N accumulation of shoot, root, and whole plant was significantly greater in the order NH_4_^+^ < NO_3_^–^ < mixed-N nutrition in the presence of Zn supply. The opposite was true, although less pronounced in the absence of Zn supply (Figure 4D). Compared with no Zn supply, nitrogen accumulation in shoot, root, and whole plant with N supply was increased by 13.6–45.8%, −9.9–44.0% (root N accumulation reduced with NH_4_^+^ supply), and 10.8–45.6% by Zn supply, respectively. The magnitude of increase in N accumulation of those tissues was consistently the greatest with mixed-N supply, followed by NO_3_^–^ nutrition, whereas NH_4_^+^ nutrition exhibited the lowest increase (Figure 4D).

Shoot C concentration was significantly increased, but root C concentration was not significantly affected by the three N forms compared with nil N supply, irrespective of Zn supply (Figure 4B). Among the three N forms, shoot C concentration was not significantly affected by the absence of Zn supply but was significantly decreased by NH_4_^+^ supply compared with NO_3_^–^ and mixed-N supply in the presence of Zn supply (Figure 4B). The effects of Zn supply and N forms on the C accumulation in shoot, root, and whole plant were similar to those of N in the corresponding tissues (Figure 4E).

Not only the N and Zn supplies but also their interaction had significant effects on Zn concentration and accumulation in shoot, root, and whole plants with the exception of non-significant effects of N treatments and their interactions on root Zn accumulation (Supplementary Table 3). For Zn concentration, compared with nil N supply, the three N forms significantly decreased Zn concentration of both shoot and root, with the exception of Zn concentration of shoot receiving NH_4_^+^ nutrition in the presence of Zn supply. Among the three N forms, Zn concentration in both shoot and root was significantly more improved in plants grown with NH_4_^+^ than NO_3_^–^ and mixed-N nutrition in the presence of Zn supply, whereas it was not significantly affected by the three N forms in the absence of Zn supply (Figure 4C). Total Zn accumulation including shoot and root was gradually increased in the order NO_3_^–^ < mixed-N < NH_4_^+^ nutrition in the presence of Zn supply, but not affected by the three N forms in the absence of Zn supply. As expected, irrespective of N treatments, compared with no Zn supply, Zn supply resulted in 137.0–387.4, 44.4–206.8, and 108.3–342.4% higher Zn accumulation in shoot, root, and whole plant, respectively (Figure 4F).

The proportions of shoot to total accumulation of N and Zn were not affected by the three N forms and Zn supply. The proportions of shoot to total C accumulation were improved by Zn supply, but were not significantly affected by the three N forms. In general, compared with no N application, N application significantly increased the proportions of shoot to total accumulation from 67.4 to 90.4% for N, from 54.4 to 79.1% for C, and from 51.1 to 83.5% for Zn (Supplementary Figure 1).

### The Ratios of C/N and Zn/N of Shoot and Root

**FIGURE 5:**
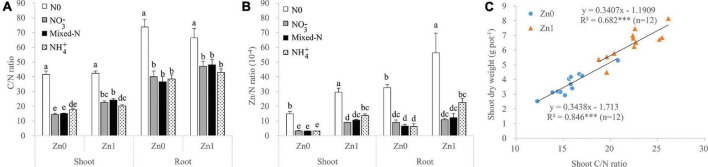
The ratios of C/N **(A)** and Zn/N **(B)** of shoot and roots of plants grown in different N forms without and with Zn supply together with the relationship between shoot C/N ratio and shoot dry weight of plants supplied with different N forms (excluding nil N treatment) without and with Zn supply **(C)**. Values are mean and SE (*n* = 4). Significant differences at *p* < 0.05 are shown with different letters. *** indicate significant difference at 0.001 level.

Among the three N forms, shoot C/N ratio was the highest with NH_4_^+^ nutrition in the absence of Zn supply, but it was the highest with mixed-N nutrition in the presence of Zn supply. Root C/N ratio was not significantly affected by the three N forms and Zn supply (Figure 5A). The Zn/N ratios of both shoot and root were not significantly affected by the three N forms without Zn supply, but those tended to increase in the order of NO_3_^–^ < mixed-N < NH_4_^+^ nutrition in the presence of Zn supply (Figure 5B). Irrespective of Zn supply, there was a significant positive correlation between shoot C/N ratio and shoot DW, implying that shoot C/N was an important indicator of shoot DW accumulation with N supply (Figure 5C).

### Other Nutrient Accumulation of Shoot and Root

Two-way ANOVA revealed significant effects of the Zn supply on shoot accumulation of Fe, Mn, K, P, Mg, and Ca (but not Cu), and significant effects of N supply on all these nutrients. The interactions between Zn and N supply were also significant for shoot accumulation of these nutrients with the exception of Ca (Supplementary Table 3). However, for root nutrient accumulation, only root accumulation of Cu and Fe was significantly affected by Zn and N supply, respectively. The interactions between Zn and N supply were also significant for root Fe accumulation (Supplementary Table 3).

**FIGURE 6:**
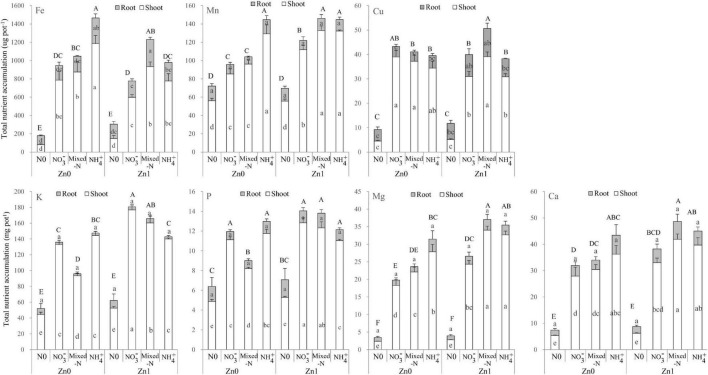
Other nutrient (Fe, Mn, Cu, K, P, Mg, and Ca) uptakes of shoot and roots of plants grown in different N forms without and with Zn supply. Values are mean and SE (*n* = 4). Significant differences at *p* < 0.05 are shown with different letters.

Figure 6 shows that under a Zn-deficient condition, total and shoot accumulation of Fe, Mn, Mg, and Ca was the highest with NH_4_^+^ nutrition, followed by mixed-N nutrition, whereas NO_3_^–^ nutrition exhibited the lowest among the three N forms. For both K and P, total and shoot accumulation tended to decrease in the order NH_4_^+^ > NO_3_^–^ > mixed-N nutrition. Under a Zn-sufficient condition, total and shoot accumulation of Fe, Mn, Mg, and Ca was the highest with mixed-N nutrition, followed by NH_4_^+^ nutrition, whereas NO_3_^–^ nutrition exhibited the lowest among the three N forms. For both K and P, total and shoot accumulation tended to increase in the order NH_4_^+^ < mixed-N < NO_3_^–^ nutrition. There were also significant and positive effects of Zn supply on shoot accumulation of Mg and Ca with each N form, and of Mn, K, and P with NO_3_^–^ and mixed-N nutrition. However, compared with no Zn supply, shoot Fe accumulation was significantly lower with Zn supply when grown with NH_4_^+^ nutrition (Figure 6).

## Discussion

Previous studies have shown that co-provision of NO_3_^–^ and NH_4_^+^ stimulates plant growth beyond that observed with either N source provided individually in several crops (Gentry and Below, 1993; Marschner, 2011; Hachiya and Sakakibara, 2017; Cao et al., 2018; Wang et al., 2019). Furthermore, fertilizer Zn supply also improves shoot biomass accumulation and the final grain yield of maize (Sarwar et al., 2017; Zhang J. Y. et al., 2019; Liu et al., 2020) and wheat (Liu et al., 2019). However, the interaction between N forms and Zn nutritional status on plant growth has received considerably less attention. In this study, our results suggested that N regimes (including N levels and N forms), Zn supply, and their interactions exerted a significant effect on the growth of maize seedlings.

The extent of the effects of Zn supply on the DW of both shoot and root and N uptake depended on N availability in the growth medium. When N was deficient in solution, Zn supply had little effects on the growth and N absorption of maize seedlings, in agreement with others (Kutman et al., 2011b). However, when N was applied at a recommended rate, the positive effects of Zn supply on the growth and N absorption of maize seedlings were dependent on N forms. Generally, the growth and N absorption of maize seedlings supplied with NH_4_^+^ supply were superior to either NO_3_^–^ or mixed-N nutrition under Zn-deficient conditions. However, the growth and N absorption of maize seedlings supplied with mixed-N supply were superior to independent NH_4_^+^ and NO_3_^–^ nutrition under Zn-sufficient conditions. Furthermore, the positive effects of mixed-N nutrition and Zn supply on the growth of maize seedlings were more than additive. For example, with zero Zn supply, NH_4_^+^ supply resulted in 374.2% of increase in shoot DW, and with zero N supply, Zn supply increased the shoot DW by 22.4%, but the combined effects of mixed-N nutrition and Zn supply were over 700% (Table 1). Similar results were also found for N absorption in the shoot, root, and whole plant (Figure 4D). Similarly, a previous study also reported that the positive effects of high N and Zn supply on the grain yield of wheat were more than additive under greenhouse conditions (Kutman et al., 2011a). For example, at low Zn supply, high N supply resulted in a 73% increase in grain yield, and at low N supply, high Zn supply increased the grain yield by 35%, but the combined effect of high N–high Zn treatment was over 350%. Previous results obtained from greenhouse conditions showed Zn and N are synergistic in their effects on increasing plant Zn concentration, and their levels in growth medium should be at enough levels to achieve the synergistic effect of N on wheat root Zn uptake (Kutman et al., 2010, 2011a,b). Here, our results showed that compared with no N supply, a substantial increase in DW of shoot and root by N supply resulted in a significant decrease in Zn concentration (a so-called dilution effect) with the exception of Zn concentration in shoots of plants receiving both NH_4_^+^ and Zn supply (Table 1 and Figure 4C). Finally, the total Zn accumulation, including shoot and root, was significantly increased by NH_4_^+^ supply in the presentence of Zn supply, but not significantly affected by N rates and forms in the absence of Zn supply. These results suggested that the combined applications of NH_4_^+^ and Zn fertilizers have beneficial effects on plant Zn nutrition. The NH_4_^+^-induced acidification and a decline in apoplastic pH may play an important role in increasing Zn availability in solution for root uptake. The abundance of root Zn uptake transporters in the plasma membrane of root cells, including ZIPs such as IRT1 and other unknown proteins (Ishimaru et al., 2005; Palmer and Guerinot, 2009; Tiong et al., 2014), may also be enhanced by NH_4_^+^ supply.

Zinc is an essential micro-nutrient for plants. As expected, Zn-deficient stress resulted in a 21.1–55.7% of decrease in shoot DW and 0.5–47.9% of decrease in root DW irrespective of N forms. These results revealed that shoots were more sensitive to Zn deficiency than the roots, in agreement with previous studies on Zn deficiency in maize (Wang and Jin, 2005; Zhang J. Y. et al., 2019). Zn deficiency causes visible symptoms when Zn content is below 15–20 mg kg^–1^ in plants (Marschner, 2011; Zhang J. Y. et al., 2019). In this study, under Zn-deficient conditions, shoot Zn concentration significantly decreased from 14.6 mg kg^–1^ to around 8.7 mg kg^–1^ with N supply (Figure 4C). These values were below the critical Zn-deficient range of 15–20 mg kg^–1^ (Marschner, 2011), indicating a Zn-deficient status although severe Zn-deficient symptoms did not occur in maize seedlings (Supplementary Figure 2). The reduced shoot Zn concentration by N supply without causing severe Zn-deficient symptoms in maize seedlings may also indicate that adequate N application improves Zn mobility and physiological availability at the cellular level by affecting the level of Zn-chelating compounds, such as amino acids, peptides, or nicotianamine (Kutman et al., 2010). Alternatively, Denghai605 may be a highly Zn-efficient cultivar with ZE values of shoot ad root being 53.9 and 69.5%. However, the corresponding ZE values of shoot and root were only 36.8 and 50.0% for Zhengdan958 grown in Zn-free nutrient solution, which showed severely Zn-deficient symptoms 15 days after treatment (Zhang J. Y. et al., 2019). According to the criteria suggested by Farquhar and Sharkey (1982), when the *P*_*n*_ decreases along with an increase in *C*_*i*_, photosynthesis is mainly limited by non-stomatal factors. In this study, compared with Zn supply, Zn deficiency resulted in a slight decrease in *P*_*n*_, whereas *C*_*i*_ was significantly increased with *g*_*s*_ and *T*_*r*_ not being affected regardless of N forms. Therefore, the reduction in *P*_*n*_ caused by Zn deficiency was mainly due to non-stomatal factors. The decreased *P*_*n*_ resulting from Zn deficiency may be due to the decline in the activities of carbonic anhydrase (Ohki, 1978), ribulose 1,5-bisphosphate carboxylase/oxygenase and fructose-1,6-bisphosphase (Hacisalihoglu, 2003; Marschner, 2011), and the dramatic damages of chloroplast structure and functions (Chen et al., 2010; Zhang J. Y. et al., 2019) as shown by the decreasing leaf SPAD values (Figure 1A).

Under Zn-deficient stress, the DW of both shoot and root was the highest with NH_4_^+^ nutrition among the three N forms. Furthermore, ZE of both shoot and root was significantly higher with NH_4_^+^ nutrition compared with NO_3_^–^ and mixed-N supply (Table 1). These results suggested that NH_4_^+^ nutrition enhanced the tolerance of maize seedlings to Zn-deficient stress. Similarly, other studies also showed the preference of growth and N uptake for NH_4_^+^ over NO_3_^–^ in maize (Gu et al., 2013; George et al., 2016; Zhang H. Q. et al., 2019). Several possible reasons are suggested for the growth preference of maize supplied by NH_4_^+^ over either NO_3_^–^ or mixed-N nutrition under Zn-deficient conditions. First, NH_4_^+^ supply produced the highest FRL, MRL, and thick roots and finally resulted in the highest TRL and total root surface area among the three N forms in the absence of Zn supply (Figure 3). The root proliferation with NH_4_^+^ supply was beneficial to promote the root nutrient uptake such as N, P, K, and especially Fe, Mn, Mg, and Ca accumulation (Figures 4, 6) and thus to improve crop growth. Similarly, a previous study also reported that the root density and extension of maize seedlings were greater in nutrient solutions containing NH_4_^+^ than in those containing NO_3_^–^ nutrition due to more rapid cell division in the root apical meristem of maize under NH_4_^+^ nutrition (Bloom et al., 2002). Results from field experiments also showed that the localized application of NH_4_-N + P significantly improved maize root biomass, the TRL, and lateral root proliferation at the seedling stage compared with localized application of NO_3_-N + P and urea +P, which could greatly contribute to improved nutrient uptake and biomass accumulation, and thus improve grain yield of maize grown in the calcareous soil with a critical Zn deficiency (DTPA-extractable Zn was 0.65 mg kg^–1^) (Ma et al., 2014). Furthermore, the NH_4_^+^-induced acidification and a decline in apoplastic pH cannot be ruled out as playing a role in increasing nutrient availability (and hence uptake), especially for Fe, Mn, Mg, and Ca uptake even though the pH of the nutrient solution was maintained at 6.0. More importantly, the uptake and assimilation of NH_4_^+^ by maize is a more energy-efficient process than that of NO_3_^–^ because the reduction of each nitrate molecule to ammonium consumes about 15 ATP molecules, which are not consumed when ammonium is supplied (Salsac et al., 1987). Less energy is needed for roots to take up and assimilate NH_4_^+^ may be more adaptive under Zn-deficient stress. Additionally, the leaf protein content with NH_4_^+^ nutrition was significantly improved by Zn deficiency compared with Zn supply and was also slightly higher when compared to NO_3_^–^ and mixed-N nutrition under Zn-deficient conditions (Figure 1B), which can prevent the dehydration of cells and enhanced the structure and function of cell membranes in adverse environments (Andrews et al., 2005; Cao et al., 2018), such as in Zn-deficient stress. Other studies reported that the supply of NH_4_^+^ nutrition significantly enhanced the drought tolerance of rice seedlings compared with the application of NO_3_^–^, and this effect seems to be closely related to the larger root tips and surface area, higher chlorophyll and Rubisco contents, and higher Rubisco activity due to the higher distribution of N absorbed to Rubisco (Guo et al., 2007; Gao et al., 2010; Ding et al., 2015). This information may be useful for enabling integrated management of Zn-deficient soils and increasing plant and grain production in the future.

Under Zn-sufficient conditions, the DW of shoot, root, and whole plant tended to increase in the order NH_4_^+^ < NO_3_^–^ < mixed-N nutrition. Compared with NH_4_^+^ nutrition alone, mixed-N supply resulted in a 27.4 and 28.1% increase in leaf *P*_*n*_ and *g*_*s*_, which further resulted in a 35.7 and 33.5% increase in shoot C accumulation and shoot DW, respectively. Furthermore, mixed-N supply resulted in 19.7% of higher shoot C/N ratio vs. NH_4_^+^ nutrition alone, which means a higher shoot biomass accumulation because of a significant positive correlation between shoot C/N ratio and shoot DW (Figure 5C). Additionally, compared with the mixed-N supply, the negative effect of NH_4_^+^ nutrition on the root DW was larger than that on the TRL due to insufficient C supply from shoot to root, resulting in an overall increase in the specific root length (the ratio of TRL to root DW) (Table 1 and Figure 3A). Previous studies suggested that an increase in specific root length is an adaptive response to insufficient carbohydrate (Ostonen et al., 2007; Xie et al., 2007). Wang et al. (2019) also showed that compared with NH_4_^+^ nutrition alone, mixed-N nutrition resulted in a significantly increased shoot and root biomass mainly due to increased shoot ATP content and greater leaf area with similar or even lower *P*_*n*_ under lower and higher planting densities. Other studies also reported that mixed-N nutrition tended to increase both the accumulation of whole-shoot DW and the proportion partitioned to reproductive tissues and finally to increase the grain yield of maize, compared to NO_3_^–^ nutrition only (Gentry and Below, 1993). Another field experiment showed that maize grain and straw yields were highest when fertilized with calcium ammonium nitrate, followed by ammonium sulfate, whereas urea exhibited the lowest yields (Abbasi et al., 2013).

## Conclusion

Irrespective of N forms, compared with Zn supply, Zn deficiency resulted in a substantial decrease in both shoot and root DW, with the magnitude of decrease consistently being the highest with mixed-N nutrition, followed by NO_3_^–^ nutrition whereas NH_4_^+^ nutrition exhibited the lowest decrease. Furthermore, ZE of both shoot and root was significantly higher with NH_4_^+^ nutrition compared with NO_3_^–^ and mixed-N supply. These results suggested that NH_4_^+^ nutrition enhanced the tolerance of maize seedlings to Zn-deficient stress. Under Zn-sufficient conditions, the DW of the shoot, root, and whole plant tended to increase in the order NH_4_^+^ < NO_3_^–^ < mixed-N nutrition. Furthermore, the positive effects of mixed-N nutrition and Zn supply on the growth of maize seedlings were more than additive. The combined applications of mixed-N nutrition and Zn fertilizer can promote root growth (including the increased root DW, root length in different diameter, TRL, and total root surface area), synchronously improve root absorption capacity of N, Fe, Cu, Mn, Mg, and Ca, increase leaf SPAD values and photosynthetic rate, and thus maximize the aboveground plant dry matter accumulation. In the case of total Zn uptake, the positive effect of NH_4_^+^ nutrition with Zn supply was more pronounced. This information may be useful for enabling integrated N management of Zn-deficient and Zn-sufficient soils and increasing plant and grain production in the future.

## Data Availability Statement

The original contributions presented in the study are included in the article/Supplementary Material, further inquiries can be directed to the corresponding authors.

## Author Contributions

KL, ZL, and YX conceived and designed the experiments, analyzed the data, and wrote the manuscript. WY, YG, HZ, LJ, XQ, ZC, CZ, SL, and HW performed the experiments. All the authors read and approved the final manuscript.

## Conflict of Interest

The authors declare that the research was conducted in the absence of any commercial or financial relationships that could be construed as a potential conflict of interest.

## Publisher’s Note

All claims expressed in this article are solely those of the authors and do not necessarily represent those of their affiliated organizations, or those of the publisher, the editors and the reviewers. Any product that may be evaluated in this article, or claim that may be made by its manufacturer, is not guaranteed or endorsed by the publisher.

## Supplementary Material

Supplementary Figure 1 The proportions of shoot to total N, C, and Zn accumulation of 30-day-old maize seedlings cultured with nil N (N0), NO_3_^–^, mixed-N, and NH_4_^+^ nutrition under sand culture conditions without (Zn0) and with Zn (Zn1) supply. Error bars represent the standard error of the mean (*n* = 4). Significant differences at *p* < 0.05 are shown with different letters.

Supplementary Figure 2 The growth of 30-day-old maize seedlings cultured with nil N (N0), NO_3_^–^, mixed-N, and NH_4_^+^ nutrition under sand culture conditions without (Zn0) and with Zn (Zn1) supply.
