# Supplementary figures and images for: Interaction Effects of Nitrogen Rates and Forms Combined With and Without Zinc Supply on Plant Growth and Nutrient Uptake in Maize Seedlings
Source: Front Plant Sci. 2021 Dec 9;12:722752. doi: 10.3389/fpls.2021.722752 (PMC8695760; doi:10.3389/fpls.2021.722752)

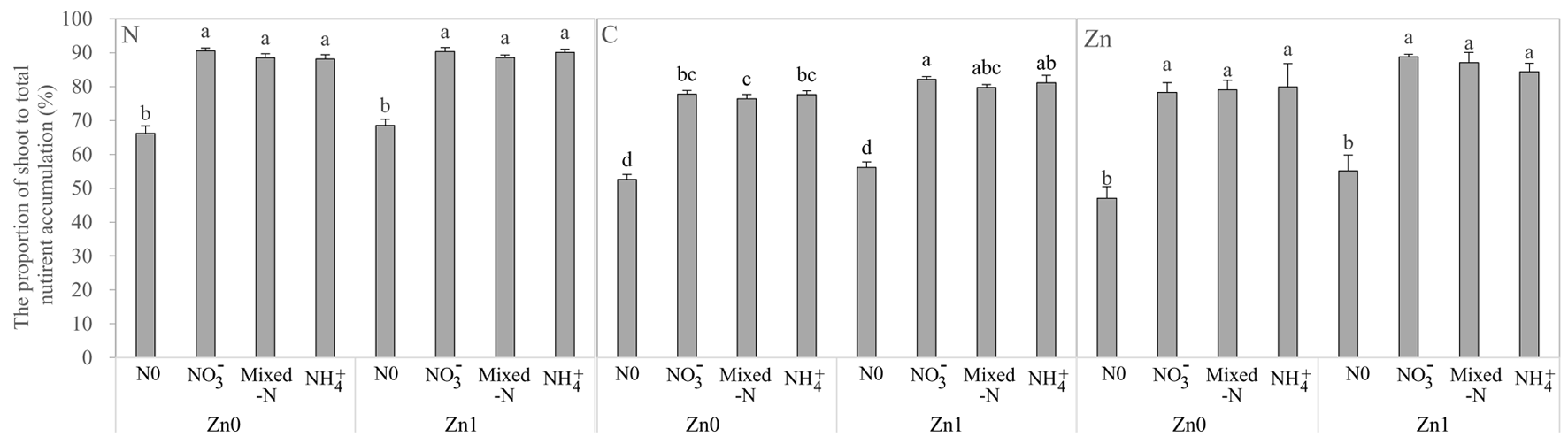

Supplement: Supplementary Figure 1 — The proportions of shoot to total N, C, and Zn accumulation of 30-day-old maize seedlings cultured with nil N (N0), NO3–, mixed-N, and NH4+ nutrition under sand culture conditions without (Zn0) and with Zn (Zn1) supply. Error bars represent the standard error of the mean (n = 4). Significant differences at p < 0.05 are shown with different letters. [file Image_1.TIF]

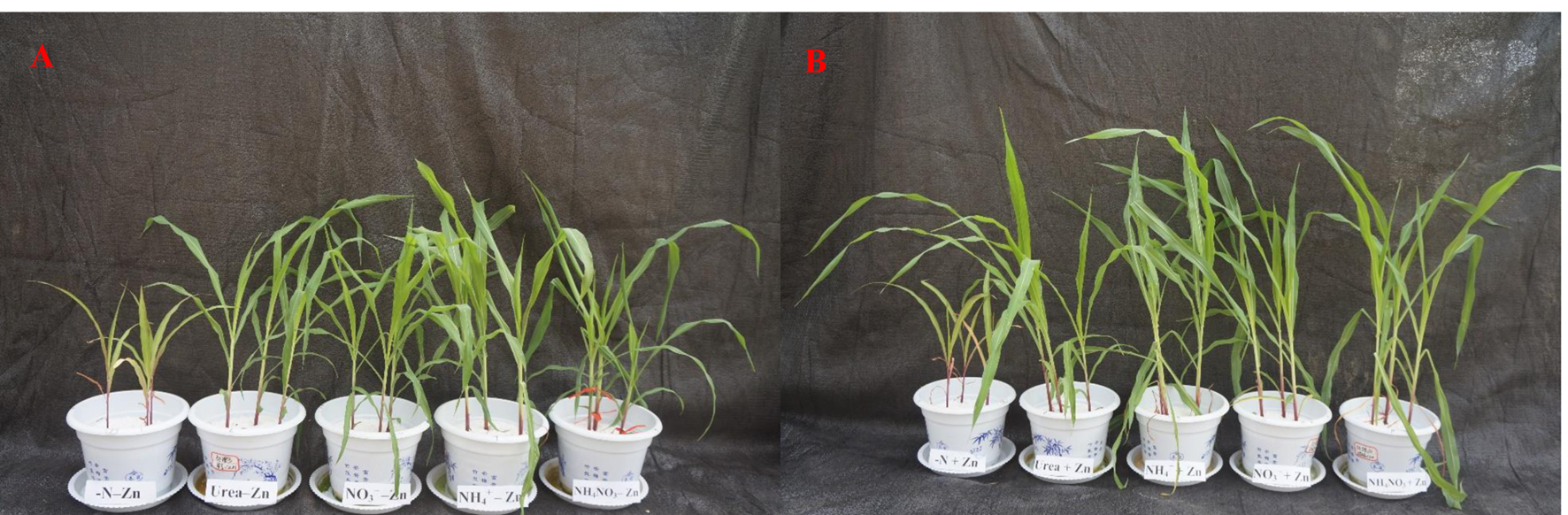

Supplement: Supplementary Figure 2 — The growth of 30-day-old maize seedlings cultured with nil N (N0), NO3–, mixed-N, and NH4+ nutrition under sand culture conditions without (Zn0) and with Zn (Zn1) supply. [file Image_2.TIF]
