# Supplementary material for: Interaction Effects of Nitrogen Rates and Forms Combined With and Without Zinc Supply on Plant Growth and Nutrient Uptake in Maize Seedlings
Source: Front Plant Sci. 2021 Dec 9;12:722752. doi: 10.3389/fpls.2021.722752 (PMC8695760; doi:10.3389/fpls.2021.722752)
Supplement: Supplementary file 3 [file Table_1.docx]

**Supplementary materials**

**TABLE S1** Two-way analysis of variance (ANOVA) of the effects of N supply, Zn supply, and their interactions on the leaf SPAD values, soluble protein contents, *P_n_*, *C_i_*. *g_s_* and *T_r_*_._

| Source of variation | DF | SPAD | |  | Soluble protein content | |  | *P_n_* | |  | *C_i_* | |  | *g_s_* | |  | *T_r_* | |
| --- | --- | --- | --- | --- | --- | --- | --- | --- | --- | --- | --- | --- | --- | --- | --- | --- | --- | --- |
|  |  | SS | F Pr. |  | SS | F Pr. |  | SS | F Pr. |  | SS | F Pr. |  | SS | F Pr. |  | SS | F Pr. |
| N | 3 | 990.01 | 0.0001 |  | 7.11 | 0.0001 |  | 341.02 | 0.0001 |  | 2779.58 | 0.3852 |  | 0.02 | 0.0001 |  | 5.30 | 0.0001 |
| Zn | 1 | 61.30 | 0.0003 |  | 0.07 | 0.1564 |  | 61.72 | 0.0018 |  | 30400.85 | 0.0001 |  | 0.00 | 0.5990 |  | 0.00 | 0.9538 |
| N*Zn | 3 | 3.56 | 0.7856 |  | 1.44 | 0.0001 |  | 21.85 | 0.2399 |  | 9373.02 | 0.0308 |  | 0.00 | 0.5348 |  | 0.11 | 0.7430 |

**TABLE S2** Two-way analysis of variance (ANOVA) of the effects of N supply, Zn supply, and their interactions on the fine root length (FRL), medium-sized root length (MRL), thick root length, total root length (TRL) and the proportions of root length in different diameters to TRL.

| Source of Variation | DF |  | Fine root length (FRL) | |  | Medium-sized root length (MRL) | |  | Thick root length | |  | Total root length (TRL) | |
| --- | --- | --- | --- | --- | --- | --- | --- | --- | --- | --- | --- | --- | --- |
|  |  |  | SS | F Pr. |  | SS | F Pr. |  | SS | F Pr. |  | SS | F Pr. |
| N treatment (N) | 3 |  | 25148884 | 0.0046 |  | 9460411 | 0.0049 |  | 1957370 | 0.032 |  | 88462429 | 0.0038 |
| Zn treatment (Zn) | 1 |  | 6225784 | 0.0412 |  | 2320501 | 0.0431 |  | 477017 | 0.1128 |  | 22175803 | 0.0358 |
| N*Zn | 3 |  | 2311716 | 0.6103 |  | 1070698 | 0.5344 |  | 784223 | 0.2413 |  | 11344348 | 0.4566 |
| Source of Variation | DF |  | The proportion of FRL to TRL | |  | The proportion of MRL to TRL | |  | The proportion of thick root length to TRL | |  |  | |
|  |  |  | SS | F Pr. |  | SS | F Pr. |  | SS | F Pr. |  |  |  |
| N treatment (N) | 3 |  | 2.6094 | 0.966 |  | 129.4219 | 0.0117 |  | 156.1846 | 0.0101 |  |  |  |
| Zn treatment (Zn) | 1 |  | 0.0234 | 0.9621 |  | 23.0104 | 0.1141 |  | 24.3815 | 0.1291 |  |  |  |
| N*Zn | 3 |  | 10.2083 | 0.7967 |  | 14.385 | 0.6306 |  | 23.204 | 0.5016 |  |  |  |

**TABLE S3** Two-way analysis of variance (ANOVA) of the effects of N supply, Zn supply, and their interactions on the nutrient accumulation including N, C, Zn, Fe, Mn, Cu, K, P, Mg and Ca in shoot, root and total plant cultured with nil N (N0), NO_3_^–^, mixed-N and NH_4_^+^ nutrition under sand culture conditions without (Zn0) and with Zn (Zn1) supply.

| Parameter | Source of variation | DF | N | |  | C | |  | Zn | |  | Fe | |  | Mn | |
| --- | --- | --- | --- | --- | --- | --- | --- | --- | --- | --- | --- | --- | --- | --- | --- | --- |
|  |  |  | SS | F Pr. |  | SS | F Pr. |  | SS | F Pr. |  | SS | F Pr. |  | SS | F Pr. |
| Shoot concentration | Zn | 1 | 253.13 | 0.0001 |  | 10.53 | 0.0001 |  | 1398.15 | 0.0001 |  | 68075.89 | 0.0001 |  | 676.38 | 0.0001 |
|  | N | 3 | 1104.33 | 0.0001 |  | 21.30 | 0.0001 |  | 115.58 | 0.0001 |  | 46888.38 | 0.0001 |  | 6071.79 | 0.0001 |
|  | Zn*N | 3 | 150.51 | 0.0001 |  | 2.68 | 0.0009 |  | 65.98 | 0.0011 |  | 48249.95 | 0.0001 |  | 51.98 | 0.2794 |
| Root concentration | Zn | 1 | 18.76 | 0.0098 |  | 0.03 | 0.9238 |  | 594.44 | 0.0008 |  | 1687.51 | 0.3693 |  | 66.41 | 0.1134 |
|  | N | 3 | 142.23 | 0.0001 |  | 2.03 | 0.8600 |  | 1634.54 | 0.0001 |  | 11876.81 | 0.1486 |  | 638.38 | 0.0006 |
|  | Zn*N | 3 | 11.57 | 0.2064 |  | 0.32 | 0.9892 |  | 381.12 | 0.0409 |  | 21142.46 | 0.0330 |  | 7.10 | 0.9607 |
| Shoot accumulation | Zn | 1 | 3318.27 | 0.0001 |  | 7166141 | 0.0933 |  | 53953.66 | 0.0001 |  | 110212.77 | 0.0290 |  | 2180.64 | 0.0001 |
|  | N | 3 | 60518.53 | 0.0001 |  | 18239882 | 0.1092 |  | 23613.69 | 0.0001 |  | 3680720.30 | 0.0001 |  | 24814.49 | 0.0001 |
|  | Zn*N | 3 | 1677.80 | 0.0017 |  | 3639296 | 0.0001 |  | 12782.19 | 0.0001 |  | 311023.15 | 0.0078 |  | 1951.18 | 0.0025 |
| Root accumulation | Zn | 1 | 24.57 | 0.0859 |  | 187456 | 0.0004 |  | 1226.24 | 0.0002 |  | 7591.58 | 0.1257 |  | 0.01 | 0.9894 |
|  | N | 3 | 440.58 | 0.0001 |  | 301586 | 0.0003 |  | 471.78 | 0.0804 |  | 72585.88 | 0.0009 |  | 140.55 | 0.3752 |
|  | Zn*N | 3 | 49.29 | 0.1216 |  | 186729 | 0.0041 |  | 226.02 | 0.3210 |  | 42040.49 | 0.0116 |  | 81.14 | 0.6044 |
| Total accumulation | Zn | 1 | 3914.13 | 0.0001 |  | 9671862 | 0.0001 |  | 71442.00 | 0.0001 |  | 59951.46 | 0.1547 |  | 2172.39 | 0.0009 |
|  | N | 3 | 70953.76 | 0.0001 |  | 22984859 | 0.0001 |  | 20440.15 | 0.0001 |  | 4729505.29 | 0.0001 |  | 23515.79 | 0.0001 |
|  | Zn*N | 3 | 2219.00 | 0.0018 |  | 5382321 | 0.0001 |  | 14196.37 | 0.0001 |  | 568935.13 | 0.0021 |  | 2745.96 | 0.0032 |
|  |  |  | Cu | |  | K | |  | P | |  | Mg | |  | Ca | |
|  |  |  | SS | F Pr. |  | SS | F Pr. |  | SS | F Pr. |  | SS | F Pr. |  | SS | F Pr. |
| Shoot concentration | Zn | 1 | 120.01 | 0.0001 |  | 412.20 | 0.0001 |  | 6.12 | 0.0001 |  | 8.97 | 0.0001 |  | 32.26 | 0.000 |
|  | N | 3 | 67.63 | 0.0001 |  | 2227.66 | 0.0001 |  | 43.01 | 0.0001 |  | 64.75 | 0.0001 |  | 22.40 | 0.000 |
|  | Zn*N | 3 | 55.88 | 0.0001 |  | 115.10 | 0.1270 |  | 0.61 | 0.2522 |  | 5.44 | 0.0001 |  | 14.46 | 0.000 |
| Root concentration | Zn | 1 | 33.74 | 0.0084 |  | 3.35 | 0.8520 |  | 0.02 | 0.9108 |  | 0.08 | 0.8685 |  | 0.78 | 0.7558 |
|  | N | 3 | 16.80 | 0.2690 |  | 322.88 | 0.3534 |  | 7.10 | 0.2453 |  | 6.65 | 0.5347 |  | 19.58 | 0.4891 |
|  | Zn*N | 3 | 0.17 | 0.9977 |  | 13.08 | 0.9863 |  | 0.27 | 0.9821 |  | 0.31 | 0.9910 |  | 3.33 | 0.9336 |
| Shoot accumulation | Zn | 1 | 40.82 | 0.1040 |  | 6534.82 | 0.0001 |  | 15.28 | 0.0001 |  | 268.81 | 0.0001 |  | 217.26 | 0.004 |
|  | N | 3 | 5684.53 | 0.0001 |  | 54904.94 | 0.0001 |  | 237.94 | 0.0001 |  | 3795.65 | 0.0001 |  | 5321.11 | 0.000 |
|  | Zn*N | 3 | 119.32 | 0.0641 |  | 6434.80 | 0.0001 |  | 26.02 | 0.0001 |  | 134.33 | 0.0023 |  | 123.35 | 0.156 |
| Root accumulation | Zn | 1 | 136.87 | 0.0004 |  | 15.72 | 0.6742 |  | 0.76 | 0.4761 |  | 1.12 | 0.6613 |  | 4.56 | 0.5972 |
|  | N | 3 | 18.77 | 0.4954 |  | 161.21 | 0.6092 |  | 1.89 | 0.7277 |  | 18.48 | 0.3772 |  | 68.05 | 0.2615 |
|  | Zn*N | 3 | 42.13 | 0.1693 |  | 10.68 | 0.9886 |  | 0.63 | 0.9306 |  | 5.60 | 0.8048 |  | 26.72 | 0.6457 |
| Total accumulation | Zn | 1 | 28.18 | 0.3237 |  | 7191.30 | 0.0001 |  | 22.85 | 0.0043 |  | 304.67 | 0.0001 |  | 284.89 | 0.0190 |
|  | N | 3 | 6222.61 | 0.0001 |  | 49209.45 | 0.0001 |  | 197.98 | 0.0001 |  | 4295.99 | 0.0001 |  | 6551.75 | 0.0001 |
|  | Zn*N | 3 | 190.90 | 0.1061 |  | 6799.89 | 0.0002 |  | 34.41 | 0.0080 |  | 178.65 | 0.0128 |  | 234.77 | 0.1830 |
